# Factors associated with HIV status non-disclosure among people entering care at IeDEA sites in Cameroon: a cross-sectional study

**DOI:** 10.1186/s12981-025-00765-1

**Published:** 2025-10-08

**Authors:** Gabriel Tchatchouang Mabou, Ellen Brazier, Peter Ebasone, Anastase Dzudie, Donald Hoover, Qiuhu Shi, Ryan Barthel, Rogers Ajeh, Denis Nsame Nforniwe, Annereke Nyenti, Joseph Mendimi Nkodo, Denis Nash, Adebola Adedimeji, Marcel Yotebieng, Kathryn Anastos

**Affiliations:** 1 Clinical Research Education Networking and Consultancy (CRENC), Yaounde, Cameroon; 2 Institute for Implementation Science in Population Health, Graduate School of Public Health and Health Policy, City University of New York (CUNY), New York, USA; 3 Faculty of Medicine and Biomedical Sciences, University of Yaounde I, Yaounde, Cameroon; 4 Service of Internal Medicine, Douala General Hospital, Douala, Cameroon; 5 Department of Global Health and Population, Lown Scholars Program, Harvard T.H. Chan School of Public Health, Boston, MA USA; 6 Department of Statistics and Institute for Health, Health Care Policy and Aging Research, Rutgers the State University of New Jersey, Piscataway, NJ USA; 7 Department of Public Health, New York Medical College, Valhalla, New York USA; 8 Limbe Regional Hospital, Limbe, Cameroon; 9 Bamenda Regional Hospital, Bamenda, Cameroon; 10 Yaounde Jamot Hospital, Yaounde, Cameroon; 11 Department of Morphological Sciences and Pathological Anatomy, Faculty of Medicine and Biomedical Sciences, University of Yaounde I, Yaounde, Cameroon; 12 Department of Epidemiology and Biostatistics, Graduate School of Public Health, City University of New York, New York, NY USA; 13 Department of Epidemiology & Population Health, Albert Einstein College of Medicine, Bronx, NY USA; 14 Department of Medicine, Albert Einstein College of Medicine, Bronx, NY USA; 15 Departments of Medicine and Epidemiology & Population Health, Albert Einstein College of Medicine, Bronx, NY USA

**Keywords:** Cameroon, HIV status disclosure, Patients living with HIV, IeDEA

## Abstract

**Background:**

While non-disclosure of HIV status may protect people living with HIV (PLWH) against stigma, discrimination, and violence, disclosure may facilitate access to social support and improve treatment adherence. This study examined factors associated with non-disclosure among recently-diagnosed PLWH at IeDEA study sites in Cameroon.

**Methods:**

We conducted a cross-sectional study of adults ≥ 19 years newly enrolling in HIV care at three Cameroon hospitals from January 2016 to June 2023 with recent (< 1 year) diagnoses and no evidence of prior HIV care. We used logistic regression to identify factors associated with non-disclosure of HIV status at the time of enrolment.

**Results:**

Among 2880 participants, the overall prevalence of HIV status non-disclosure at enrolment was 34.4%, ranging from 48.0% among those enrolling on the day of diagnosis to 18.7% among those enrolling > 30 days after diagnosis. Men and single participants had higher odds of non-disclosure compared with women (aOR: 1.68; 95% CI 1.38, 2.04) and those who were married/living with a partner (aOR: 1.66; 95% CI 1.36, 2.02). Those with early-stage HIV disease (WHO Stage 1 or 2 or CD4 ≥ 200 cells/mm^3^) also had higher odds of non-disclosure (aOR: 1.48; 95% CI 1.20, 1.83) compared with participants with advanced-stage disease.

**Conclusion:**

Among those diagnosed with HIV within 1 year prior to enrolment, men, single/unmarried people, and those with early-stage HIV disease were less likely to disclose their status. Further research on barriers to status disclosure among these groups is needed to guide disclosure support and counselling interventions.

**Supplementary Information:**

The online version contains supplementary material available at 10.1186/s12981-025-00765-1.

## Introduction

The World Health Organization (WHO) defines HIV status disclosure as informing persons or regulatory authorities of an individual’s serostatus [1]. This process, which may involve revealing one’s HIV status to sexual partner(s), family members, friends, relatives or other individuals within a person’s social network (such as teachers, healthcare workers, religious authorities or colleagues), generally occurs progressively over time [2]. While disclosing HIV status is challenging for many people living with HIV (PLWH), research has shown that status disclosure has numerous benefits related to patient engagement in care [3, 4], initiation and adherence to ART [5–7], partner HIV testing [3], condom negotiation and use [8, 9], and social support, self-esteem and reduced anxiety [10–13]. However, this dual reality underscores the complexity of disclosure and the need for a nuanced understanding of its determinants.

The prevalence of HIV status disclosure varies widely across geographic and cultural contexts, as well as by the time point at which disclosure status is examined. Past studies have reported variation in HIV status non-disclosure, ranging from 8.1% to 61.0% [14–23]. Research conducted in diverse settings has highlighted various factors associated with non-disclosure of HIV status, including age, marital or relationship status, educational level, socio-economic status, advanced disease stage, access to health and support services, being part of an HIV/AIDS association, knowledge of partner HIV status, alcohol consumption and depression [2, 12, 18, 24–31].

Differences in disclosure have also been associated with perceived stigma and discrimination [2] and the time since HIV diagnosis [32–37]. While a few studies have explored aspects of HIV status disclosure in Cameroon [26, 38, 39], much of the existing research on HIV status disclosure pre-dates the introduction of universal HIV testing and treatment in Cameroon [40]. Furthermore, past studies examined disclosure among all patients, irrespective of patients’ time in HIV care, grouping together newly-diagnosed patients with those who had been living with HIV for extended periods of time [30, 31].

To complement past research, we examined the prevalence of HIV status non-disclosure and factors associated with non-disclosure among PLWH newly initiating care after the introduction of universal testing and treatment in Cameroon.

## Methods and materials

### Study design and period

This study was a cross-sectional analysis of data from three HIV clinics that participate in the Cameroon cohort of the Central Africa International epidemiology Databases to Evaluate AIDS (IeDEA) research collaboration (Cameroon IeDEA). As described elsewhere [41], Cameroon IeDEA is a prospective open cohort study which started in 2016 at three urban hospitals: Limbe Regional Hospital, Bamenda Regional Hospital, and Yaoundé Jamot Hospital.

The study collects primary data via interviews with study participants and abstracts clinical data from their medical records. Using standardized procedures, trained research assistants invite participants to enrol in the Cameroon IeDEA study during their routine HIV care visits, and those who provide written informed consent complete a study enrolment interview. Conducted by state registered nurses at each site who serve as study research assistants, the enrolment interviews explore participants’ education, employment, income, marital status, and HIV status disclosure. Study enrolment interviews also include questions about alcohol consumption, smoking and other substance use, as well as screening for depression using the two-item Patient Health Questionnaire (PHQ-2) [42]. The PHQ-2 is a validated depression screening tool that explores the extent to which someone has experienced “Little interest or pleasure in doing things” and “Feeling down, depressed or hopeless” during the previous 2 weeks. Responses to each question are scored from 0 (“Not at all”) to 3 (“Nearly every day”). Data abstracted from medical records include participants’ date of birth, HIV diagnosis date, HIV care enrolment date, CD4 test results, and WHO disease staging.

Ethical approval for the Cameroon IeDEA Study was granted by the National Ethics Committee of Research for Human Health (CNERSH) in Cameroon and the Institutional Review Board at Albert Einstein College of Medicine in USA in 2016, with annual renewals.

### Study population

The study focused on adults ≥ 19 years old who enrolled in the Cameroon IeDEA study on the same day they were newly enrolled in HIV care at one of the three study sites between January 1st, 2016 and June 30th, 2023. To focus on status of non-disclosure among those with recent HIV diagnoses, patients with evidence of HIV care or treatment prior to the date of study enrolment were considered ineligible, along with those whose HIV diagnosis date was unknown or more than 1 year (365 days) prior to study enrolment. This information was obtained from the patient’s medical records. Those with missing data on the primary outcome of interest (HIV status disclosure) were also excluded.

### Outcome measurement

The primary outcome of interest was HIV status non-disclosure at the time of enrolment in HIV care, which was determined based on whether the patient reported having shared his or her status with anyone at the time of the study enrolment interview.Those reporting that they had disclosed their HIV status to someone were asked about the type(s) of persons to whom they had disclosed their status (e.g., friend, child, sibling, parents, other family member, spouse/partner and/or others, including healthcare professionals, religious authorities, teachers or work colleagues).

### Participant characteristics

Participant socio-demographic characteristics included: age at study enrolment (categorized as 19–29, 30–39, 40–49, or 50 + years); sex (male or female); marital status (single, married/living with a partner, divorced, or widowed); highest grade of schooling/education completed (none, primary, secondary/high school, or university); employment status (employed/self-employed, unemployed, student, or retired); and monthly earnings in Central Africa (CFA) francs from main source of income (None, < 50,000, 51–100,000, or > 100,000). Other participant characteristics of interest included: interval (in days) between HIV diagnosis and HIV care enrolment (same-day, 1–7 days, 8–30 days, or > 30 days). Advanced HIV disease at the time of HIV care enrolment was defined according to WHO stages or CD4 test results (i.e., WHO stage 3 or 4 or CD4 < 200 cells/mm^3^ vs. WHO stage 1 or 2 or CD4 ≥ 200 cells/mm^3^). PHQ-2 scores ≥ 3 were considered indicative of likely depressive disorder [42]. Self-reported substance use included alcohol consumption (never, monthly, or weekly); smoking (never, former, or current); and drug use (never vs. ever).

### Statistical analysis

We used descriptive statistics to characterize study participants who reported non-disclosure of HIV status at study enrolment, overall, and stratified by time since HIV diagnosis (i.e., same-day, 1–7 days, 8–30 days, or > 30 days). We compared the distribution of patient characteristics related to education, income, marital status, HIV disease stage, drinking, smoking, and drug use among those with complete data for these characteristics and those with any missing data, and among patients with complete data, we used binomial logistic regression to examine characteristics associated with non-disclosure, overall, and stratified by time since diagnosis. These variables were selected for the regression model because of the literature review. All statistical analyses were performed using SAS 9.4 (SAS Institute, Cary, NC). P-values less than or equal to 0.05 were considered statistically significant.

## Results

### Study population

**Fig. 1:**
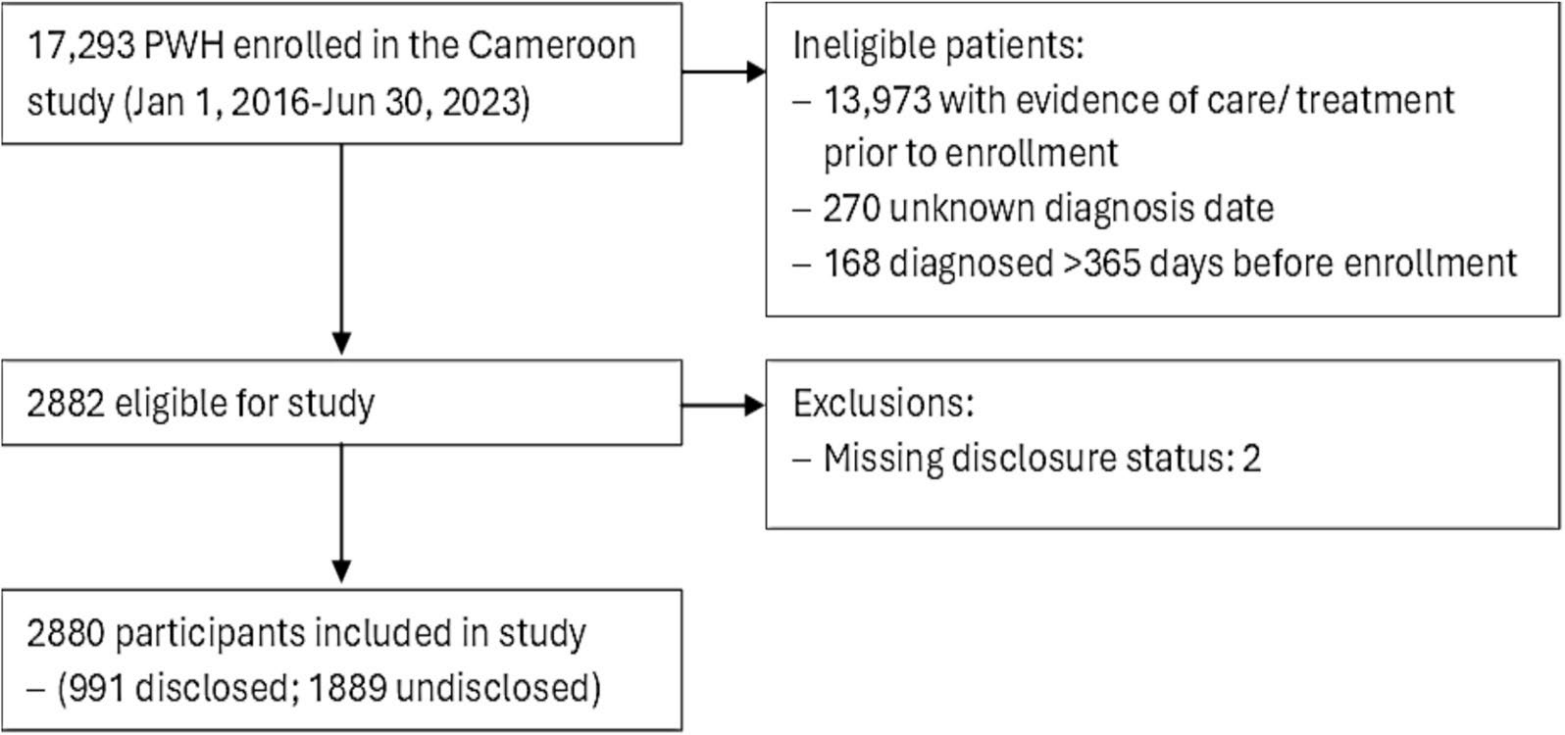
Sample flow chart of study participant selection process

A total of 17,293 PLWH enrolled in the Cameroon IeDEA study from January 1, 2016 through June 30, 2023. Of these, 2882 were eligible for inclusion in this study because they enrolled in HIV care on the same day they were enrolled in IeDEA, had no record of prior treatment or care at another HIV clinic, and had been diagnosed with HIV within the previous year. Two patients were excluded because of missing data for the outcome of interest. Details regarding subject selection are demonstrated in Fig. 1.

In our resulting sample of 2880 participants, 53.8% were female (Table 1) and median age was 36.4 years (interquartile range [IQR]: 29.2–44.7 years). Most participants reported that they were single (46.6%) or married/living with a partner (39.4%). More than one-third (40.8%) reported having any primary-level education, and a similar proportion (37.7%) reported having any secondary/high school education. Less than half (42.0%) reported that they were employed or self-employed at the time of the interview, with 28.5% reporting that they were unemployed and < 10% reporting they were currently in school or retired. Most participants reported no monthly income (34.4%) or a monthly income < 50,000 CFA (< 81.4 USD), with only one-quarter reporting a monthly income above 50,000 CFA. The majority (70.1%) had early-stage HIV disease at the time of HIV care enrolment (i.e., WHO stage 1 or 2 or CD4 cell count ≥ 200 cells/mm^3^), and 21.1% screened positive for depression (i.e., PHQ2 ≥ 3). Monthly or weekly consumption of alcohol was reported by 42.7% and 19.0%, respectively, while the large majority reported that they never smoked (81.0%) or used other drugs (98.9%).

**Table 1:** Characteristics of patients, overall, and by timing of HIV care enrolment after date of diagnosis

Characteristic	All participant N = 2880 (%)	Characteristics of participants, by days between HIV diagnosis and HIV care enrolment				
		Same day n = 1007 (34.9%)	1–7 days n = 1091 (37.9%)	8–30 days n = 531 (18.4%)	> 30 days n = 251 (8.7%)	p-value
Sex	n (col %)	n (col %)	n (col %)	n (col %)	n (col %)	0.67
Male	1331 (46.2)	468 (46.5)	514 (47.1)	241 (45.4)	108 (43.0)	
Female	1549 (53.8)	539 (53.5)	577 (52.9)	290 (54.6)	143 (57.0)	
Age group						< .001
19–29	782 (27.2)	314 (31.2)	286 (26.2)	120 (22.6)	62 (24.7)	
30–39	977 (33.9)	335 (33.3)	358 (32.8)	183 (34.5)	101 (40.2)	
40–49	720 (25.0)	217 (21.5)	284 (26.0)	151 (28.4)	68 (27.1)	
50 +	401 (13.9)	141 (14.0)	163 (14.9)	77 (14.5)	20 (8)	
Marital status						0.04
Single	1341 (46.6)	461 (45.8)	514 (47.1)	242 (45.6)	124 (49.4)	
Married/living with a partner	1135 (39.4)	427 (42.4)	411 (37.7)	201 (37.9)	96 (38.2)	
Separated/ divorced	149 (5.2)	46 (4.6)	54 (4.9)	38 (7.2)	11 (4.4)	
Widowed	243 (8.4)	66 (6.6)	108 (9.9)	50 (9.4)	19 (7.6)	
Missing/unknown	12 (0.4)	7 (0.7)	4 (0.4)	0 (0)	1 (0.4)	
Education						0.94
Never went to school	314 (10.9)	111 (11.0)	108 (9.9)	66 (12.4)	29 (11.6)	
Primary	1176 (40.8)	414 (41.1)	439 (40.2)	223 (42.0)	100 (39.8)	
Secondary/high school	1087 (37.7)	377 (37.4)	420 (38.5)	193 (36.3)	97 (38.6)	
University	287 (10.0)	100 (9.9)	116 (10.6)	47 (8.9)	24 (9.6)	
Missing/unknown	16 (0.6)	5 (0.5)	8 (0.7)	2 (0.4)	1 (0.4)	
Monthly income (CFA)						0.90
None	991 (34.4)	348 (34.6)	375 (34.4)	184 (34.7)	84 (33.5)	
< 50,000	1066 (37.0)	357 (35.5)	420 (38.5)	198 (37.3)	91 (36.3)	
51–100,000	423 (14.7)	154 (15.3)	149 (13.7)	85 (16.0)	35 (13.9)	
> 100,000	306 (10.6)	114 (11.3)	112 (10.3)	48 (9.0)	32 (12.7)	
Missing/unknown	94 (3.3)	34 (3.4)	35 (3.2)	16 (3.0)	9 (3.6)	
HIV disease stage						< .001
Early	2019 (70.1)	793 (78.7)	791 (72.5)	302 (56.9)	133 (53.0)	
Advanced	748 (26.0)	172 (17.1)	266 (24.4)	211 (39.7)	99 (39.4)	
Unknown/missing	113 (3.9)	42 (4.2)	34 (3.1)	18 (3.4)	19 (7.6)	
Depression						0.27
None (PHQ2 < 3)	2265 (78.6)	815 (80.9)	854 (78.3)	406 (76.5)	190 (75.7)	
Depressive disorder (PHQ2 ≥ 3)	608 (21.1)	189 (18.8)	235 (21.5)	123 (23.2)	61 (24.3)	
Missing/unknown	7 (0.2)	3 (0.3)	2 (0.2)	2 (0.4)	0 (0)	
Drinking						0.07
Never	1091 (37.9)	355 (35.3)	403 (36.9)	218 (41.1)	115 (45.8)	
Monthly	1229 (42.7)	435 (43.2)	477 (43.7)	219 (41.2)	98 (39.0)	
Weekly	546 (19.0)	210 (20.9)	206 (18.9)	93 (17.5)	37 (14.7)	
Missing/unknown	14 (0.5)	7 (0.7)	5 (0.5)	1 (0.2)	1 (0.4)	
Smoking						0.58
Never	2334 (81.0)	818 (81.2)	890 (81.6)	419 (78.9)	207 (82.5)	
Former	181 (6.3)	67 (6.7)	70 (6.4)	28 (5.3)	16 (6.4)	
Current	347 (12.0)	116 (11.5)	126 (11.5)	79 (14.9)	26 (10.4)	
Missing/unknown	18 (0.6)	6 (0.6)	5 (0.5)	5 (0.9)	2 (0.8)	
Drug use						0.79
Never	2847 (98.9)	998 (99.1)	1077 (98.7)	526 (99.1)	246 (98.0)	
Ever	10 (0.3)	3 (0.3)	4 (0.4)	1 (0.2)	2 (0.8)	
Missing/unknown	23 (0.8)	6 (0.6)	10 (0.9)	4 (0.8)	3 (1.2)	

Percentages are column percents. *WHO* World Health Organization. *PHQ-2* Patient Health Questionnaire-2. *CFA* Central African Franc (1 USD = 613,91 CFA, June, 2023)

Approximately one-third of our sample (34.9%) enrolled in HIV care on the day of their HIV diagnosis, with 37.9% enrolling 1–7 days after diagnosis, 18.4% enrolling 8–30 days after diagnosis, 8.4% > 30 days after diagnosis. Participant characteristics did not differ substantively by timing of enrolment after HIV diagnosis; however, higher proportions of those enrolling more than 30 days after their date of diagnosis had advanced HIV disease, compared with those enrolling in the 1st week after diagnosis.

The distribution of participant characteristics did not differ between those with complete data for all variables examined and those with any missing data (Supplementary Table 1).

### Prevalence of HIV status non-disclosure

The prevalence of HIV status non-disclosure at the time of care enrolment was (34.4%) (Table 2). Non-disclosure was higher among males, compared with females (40.3% vs. 29.3%, Chi square p < 0.001), and it was higher among study participants aged 19–29 (39.0%) compared with those 30 + years (p = 0.002) and among those who reported being single or divorced/separated, compared with those who were married or widowed (38.3% vs. 30.3%, p < 0.001). The prevalence of non-disclosure increased monotonically with education levels, ranging from 25.2% among those with no education to 40.8% among those with university-level education. The prevalence of non-disclosure was also higher among those reporting monthly income > 50,000 CFA compared with less monthly income (39.4% vs. 32.9%, p = 0.002) and among those enrolling with early-stage HIV disease, compared with those with advanced HIV disease (37.9% vs. 25.4%, p < 0.001). The prevalence of non-disclosure decreased monotonically by timing of HIV care enrolment after diagnosis, ranging from 48.0% among those enrolling on the date of diagnosis to 18.7% among those enrolling more than 30 days after diagnosis.

**Table 2:** Characteristics of patients reporting non-disclosure, overall and by timing of HIV care enrolment after date of diagnosis

Characteristic	Non-disclosure, among all participants n (%)	Non-disclosure, by timing of HIV care enrolment after date of diagnosis				
		Same day n (row %)	1–7 days n (row %)	8–30 days n (row %)	> 30 days n (row %)	p-value
All participants	991 (34.4)	483 (48.0)	354 (32.4)	107 (20.2)	47 (18.7)	
Sex						0.88
Male	537 (40.3)	260 (55.6)	197 (38.3)	55 (22.8)	25 (23.1)	
Female	454 (29.3)	223 (41.4)	157 (27.2)	52 (17.9)	22 (15.4)	
Age group						0.07
19–29	305 (39.0)	171 (54.5)	100 (35.0)	21 (17.5)	13 (21.0)	
30–39	318 (32.5)	149 (44.5)	117 (32.7)	37 (20.2)	15 (14.9)	
40–49	247 (34.3)	104 (47.9)	99 (34.9)	32 (21.2)	12 (17.6)	
50 +	121 (30.2)	59 (41.8)	38 (23.3)	17 (22.1)	7 (35.0)	
Marital status						0.08
Single	516 (38.5)	251 (54.4)	187 (36.4)	48 (19.8)	30 (24.2)	
Married/living with a partner	338 (29.8)	177 (41.5)	115 (28.0)	38 (18.9)	8 (8.3)	
Separated/divorced	54 (36.2)	24 (52.2)	18 (33.3)	9 (23.7)	3 (27.3)	
Widowed	80 (32.9)	28 (42.4)	34 (31.5)	12 (24.0)	6 (31.6)	
Missing/unknown	3 (25.0)	3 (42.9)	0 (0)	0 (0)	0 (0)	
Education						0.63
Never went to school	79 (25.2)	42 (37.8)	26 (24.1)	6 (9.1)	5 (17.2)	
Primary	409 (34.8)	196 (47.3)	144 (32.8)	53 (23.8)	16 (16.0)	
Secondary/high school	382 (35.1)	188 (49.9)	140 (33.3)	35 (18.1)	19 (19.6)	
University	117 (40.8)	56 (56.0)	42 (36.2)	13 (27.7)	6 (25.0)	
Missing/unknown	4 (2.05)	1 (20.0)	2 (25.0)	0 (0)	1 (100)	
Monthly income (CFA)						0.19
None	313 (31.6)	160 (46)	107 (28.5)	32 (17.4)	14 (16.7)	
< 50,000	363 (34.1)	178 (49.9)	126 (30.0)	46 (23.2)	13 (14.3)	
51–100,000	166 (39.2)	78 (50.6)	65 (43.6)	11 (12.9)	12 (34.3)	
> 100,000	121 (39.5)	57 (50.0)	46 (41.1)	14 (29.2)	4 (12.5)	
Missing/unknown	28 (29.8)	10 (29.4)	10 (28.6)	4 (25.0)	4 (44.4)	
HIV disease stage						< .001
Early	765 (37.9)	384(48.4)	276 (34.9)	76 (25.2)	29 (21.8)	
Advanced	190 (25.4)	73 (42.4)	71 (26.7)	29 (13.7)	17 (17.2)	
Unknown/missing	36 (31.9)	26 (61.9)	7 (20.6)	2 (11.1)	1 (5.3)	
Depression						0.06
None (PHQ2 < 3)	786 (34.7)	400 (49.1)	270 (31.6)	79 (19.5)	37 (19.5)	
Depressive disorder (PHQ2 ≥ 3)	202 (33.2)	80 (42.3)	84 (35.7)	28 (22.8)	10 (16.4)	
Missing/unknown	3 (42.9)	3 (100.0)	0 (0)	0 (0)	0 (0)	
Drinking						0.18
Never	330 (30.2)	150 (42.3)	122 (30.3)	35 (16.1)	23 (20.0)	
Monthly	446 (36.3)	216 (49.7)	162 (34.0)	49 (22.4)	19 (19.4)	
Weekly	211 (38.6)	113 (53.8)	70 (34.0)	23 (24.7)	5 (13.5)	
Missing/unknown	4 (28.6)	4 (57.1)	0 (0)	0 (0)	0 (0)	
Smoking						0.58
Never	779 (33.4)	386 (47.2)	281 (31.6)	78 (18.6)	34 (16.4)	
Former	75 (41.4)	38 (56.7)	26 (37.1)	7 (25.0)	4 (25.0)	
Current	130 (37.5)	56 (48.3)	45 (35.7)	21 (26.6)	8 (30.8)	
Missing/unknown	7 (38.9)	3/6 (50.0)	2 (40.0)	1 (20.0)	1 (50.0)	
Drug use						0.59
Never	983 (34.5)	480 (48.1)	350 (32.5)	107 (20.3)	46 (18.7)	
Ever	1 (10.0)	1 (33.3)	0 (0)	0 (0)	0 (0)	
Missing/unknown	7 (30.4)	2 (33.3)	4 (40.0)	0 (0)	1 (33.3)	

Percentages are row percents. *WHO* World Health Organization. *PHQ-2* Patient Health Questionnaire-2. *CFA* Central African Franc (1 USD = 613,91 CFA, June, 2023)

### Persons to whom status was disclosed

**Fig. 2:**
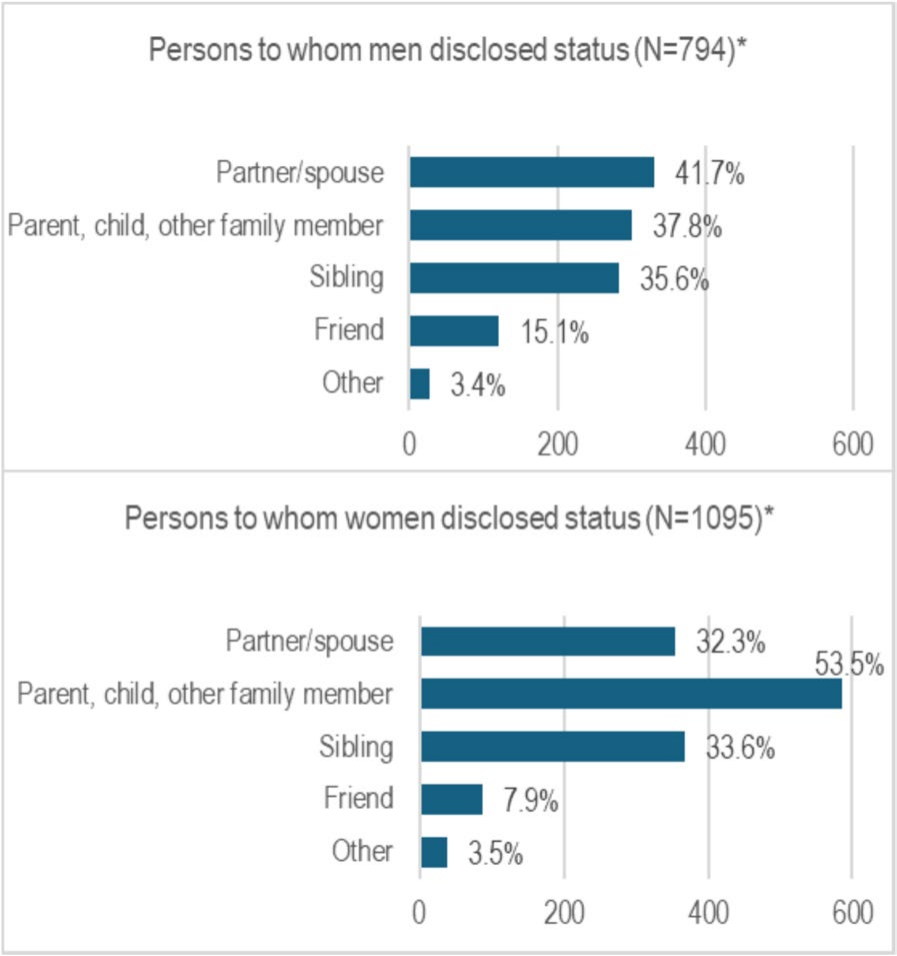
Persons to whom status was disclosed, by participant sex. *Percentages sum to > 100% because participants could report disclosure to multiple people

Among those who reported having disclosed their status, we observed differences between men and women in the persons to whom status was disclosed. This is illustrated in Fig. 2. Overall, slightly higher proportions of men reported having disclosed their HIV status to their spouses/partners, compared with women (41.7% vs. 32.3%, p < 0.001), with women predominently reporting disclosure of their status to a parent, child or other family member. Among those reporting that they had disclosed their status to only one person, similar patterns were observed, with 36.7% of men reporting that they had disclosed their status to their spouse/partner, compared with 27.5% of women (p < 0.001).

### Factors associated with HIV status non-disclosure

Table 3 reports unadjusted and adjusted odd ratios (aORs) of non-disclosure for binomial logistic regression analyses for study participants with no missing data. In our multivariable regression model, adjusting for patient characteristics (sex, age, marital status, education, income, HIV disease stage at enrolment, depression and substance use), time between HIV diagnosis and enrolment remained inversely associated with non-disclosure; the odds of non-disclosure decreased monotonically with increased time since diagnosis. Compared to those enrolling in HIV care on the date of diagnosis, the adjusted odds ratios (aOR) of non-disclosure were 0.53 (95% CI 0.43, 0.64) among those enrolling 1–7 days after diagnosis, 0.29 (95% CI 0.22, 0.37) among those enrolling 8–30 days after diagnosis, and 0.27 (95% CI 0.19, 0.39) among those enrolling > 30 days after diagnosis. Compared with women, men were more likely to report non-disclosure of their status (aOR: 1.68; 95% CI 1.38, 2.04). Those who were single (aOR: 1.66; 95% CI 1.36, 2.02), separated (aOR: 1.96; 95% CI 1.31, 2.94) or widowed (aOR: 1.77; 95% CI 1.25, 2.51) had higher odds of non-disclosure compared with married participants. Education was positively associated with non-disclosure, with odds of non-disclosure higher among those with any schooling, compared with no schooling (aOR primary schooling: 1.57, 95%CI 1.14, 2.15; aOR secondary schooling: 1.37, 95% CI 0.99, 1.91; aOR university education: 1.54, 95% CI 1.03, 2.32). Those enrolling with early-stage HIV disease had higher odds of non-disclosure (aOR: 1.48, 95% CI 1.20, 1.83), compared with participants with advanced stage disease.

**Table 3:** Factors associated with non-disclosure, among 2624 patients with no missing data related to education, income, marital status, HIV disease, drinking, smoking, and drug use

Characteristic	Unadjusted (bivariate)		Adjusted (multivariable)	
	OR (95% CI)	p-value	AOR (95% CI)	p-value
Time since diagnosis				
Same day	1		1	
1–7 days	0.54 (0.45, 0.65)	< .001	0.53 (0.43, 0.64)	< .0001
8–30 days	0.28 (0.22, 0.36)	< .0001	0.29 (0.22, 0.37)	< .0001
> 30 days	0.26 (0.18, 0.37)	< .0001	0.27 (0.19, 0.39)	< .0001
Female	1	< .0001	1	
Male	1.69 (1.44, 1.99)		1.68 (1.38, 2.04)	< .0001
Age group				
19–29	1		1	
30–39	0.79 (0.64, 0.96)	0.02	0.94 (0.74, 1.18)	0.58
40–49	0.84 (0.68, 1.05)	0.13	1.06 (0.81, 1.37)	0.68
50 +	0.67 (0.51, 0.88)	< .01	0.81 (0.58, 1.12)	0.20
Marital status				
Married/living with a partner	1		1	
Single	1.49 (1.25, 1.78)	< .0001	1.66 (1.36, 2.02)	< .0001
Separated/divorced	1.34 (0.92, 1.96)	0.13	1.96 (1.31, 2.94)	0.001
Widowed	1.13 (0.83, 1.55)	0.44	1.77 (1.25, 2.51)	0.001
Education				
Never went to school	1		1	
Primary	1.77 (1.31, 2.38)	< .001	1.57 (1.14, 2.15)	0.006
Secondary/high school	1.79 (1.33, 2.41)	< .001	1.37 (0.99, 1.91)	0.061
University	2.1 (1.46, 3.02)	< .0001	1.54 (1.03, 2.32)	0.04
Monthly income (CFA)				
None	1		1	
< 50,000 CFA	1.14 (0.95, 1.38)	0.17	1.15 (0.93, 1.41)	0.206
51–100,000 CFA	1.37 (1.08, 1.75)	0.01	1.27 (0.97, 1.66)	0.08
> 100,000 CFA	1.45 (1.11, 1.9)	0.007	1.33 (0.97, 1.81)	0.08
HIV disease stage				
Advanced	1		1	
Early	1.79 (1.48, 2.17)	< .0001	1.48 (1.20, 1.83)	< .001
Depression				
None (PHQ2 < 3)	1		1	
Depressive disorder (PHQ2 ≥ 3)	0.97 (0.79, 1.18)	0.73	1.04 (0.85, 1.29)	0.69
Drinking				
Never	1		1	
Monthly	1.35 (1.13, 1.62)	0.001	1.21 (0.99, 1.46)	0.06
Weekly	1.60 (1.28, 2.00)	< .0001	1.23 (0.96, 1.59)	0.10
Smoking				
Never	1		1	
Former	1.50 (1.09, 2.06)	0.01	0.97 (0.68, 1.39)	0.87
Current	1.24 (0.97, 1.58)	0.09	1.06 (0.80, 1.41)	0.67
Drug use				
Never	1			
Ever	0.21 (0.03, 1.64)	0.14	0.15 (0.02, 1.21)	0.075

## Discussion

In this cohort of PLWH newly enrolling in HIV care within the 1 year after diagnosis, we found that more than one-third of participants had not disclosed their HIV status. The prevalence of HIV status non-disclosure observed in our study is slightly higher than reported in earlier analyses of participants enrolled in the Cameroon IeDEA study, which have ranged from 10% to 21.1% [30, 31, 43], as well as rates of non-disclosure of 16–23% reported by studies conducted elsewhere in Africa [17, 25, 44, 45]. Differences are likely due to our focus on patients recently diagnosed with HIV and newly enrolling in care and our exclusion of patients who had been in HIV care for many years and who had more time for counselling and other support for disclosure.

Among those who do not disclose their status immediately, most appear to disclose within the month after diagnosis, and we observed relatively little change in non-disclosure after > 30 days following diagnosis. This finding agrees with other studies [34, 35, 46], that found disclosure to be positively associated with increased time since diagnosis. Our finding that there is little change in disclosure more than 2 weeks after diagnosis is particularly salient for healthcare providers. It suggests that patients’ initial follow-up visits (e.g., at 1or 2 months after care entry) may be a critical opportunity for healthcare providers to assess disclosure status, address patients’ concerns, and offer additional support and counselling to those who report no disclosure of their HIV status by the time they return for an initial follow-up visit.

Among those who disclosed, we found that HIV status was primarily disclosed to close family members (e.g., partner/spouse, parents and siblings). These findings accord with other studies [44, 47–50] which have found that PLWH most commonly disclose their status to close family members and close relatives. Consistent with other research [20, 51], we observed higher odds of HIV status non-disclosure among men compared with women. Partnership status was also strongly associated with disclosure. Participants who were single had higher odds of non-disclosure compared to those who were married or living with a partner as found in other studies [20, 27]. Disclosure may be more common among couples because of the social support afforded by these relationships and desires to prevent onward transmission of HIV [20, 27, 52, 53]. For some married/partnered PLHIV, however, non-disclosure may be protective against negative reactions (e.g., abandonment or violence, etc.) and causing distress to family members [54, 55].

We further found that the odds of non-disclosure were substantially higher among patients with early HIV disease stage at HIV care enrolment than among those with advanced-stage disease. These findings accord with Deribe et al. [27] who found that individuals with early clinical stage HIV disease had 78% lower odds of disclosure compared with those in advanced HIV disease. The authors [27] suggested that many PLWH delay disclosure until their disease has progressed, with physical symptoms becoming more noticeable making it harder to conceal the condition.

### Strengths and limitations of the study

Our robust sample size allowed for the investigation of multiple interlinked personal, relational and health-related factors in the decision not to disclose an HIV status. However, some limitations must be considered when interpreting our findings. The cross-sectional design precludes establishing causality between dependent and some of the independent variables. Secondly, most patient characteristics considered in our study were assessed via self-report, which may be prone to recall bias and social desirability biases, particularly for attributes such as education, income, and substance use. Further, while our study population was drawn from three public hospitals in Cameroon, these hospital clinics are located in large urban areas and findings may not be generalizable. Lastly, as our data do not include information about participants’ reasons for non-disclosure, qualitative research would be useful to better understand barriers to HIV disclosure.

## Conclusion

With substantial rates of non-disclosure of HIV status among Cameroonian PLWH who were newly enrolled in HIV care, our findings highlight the elevated risks of non-disclosure particularly among men, single/unmarried people, and those with early-stage HIV disease. Our finding underscore the potential value of exploring disclosure status during patient follow-up visits in order to provide additional support and counselling to those who could benefit from the social support afforded by disclosure.

## Supplementary Information

Supplementary material 1.

## Data Availability

The datasets used and/or analysed during the current study are available from the corresponding author on reasonable request. Raw data used for the analysis are availability and will be released upon request.

## Abbreviations

HIV: Human immunodeficiency virus
AIDS: Acquired immune deficiency syndrome
CA-IeDEA: Central Africa International Epidemiology Databases to Evaluate AIDS
PLWH: People living with HIV/AIDS
