# Supplementary material for: Factors associated with HIV status non-disclosure among people entering care at IeDEA sites in Cameroon: a cross-sectional study
Source: AIDS Res Ther. 2025 Oct 8;22:100. doi: 10.1186/s12981-025-00765-1 (PMC12505618; doi:10.1186/s12981-025-00765-1)
Supplement: Supplementary file 1 — Supplementary material 1. [file 12981_2025_765_MOESM1_ESM.docx]

**Supplementary Table 1. Comparison of patients with complete and missing**

| **Characteristic** | **All participants** | **Participants with complete data** | **Participants with incomplete data** | **p-value** |
| --- | --- | --- | --- | --- |
| **Time from diagnosis to enrollment** | **n=2880** | **n=2624** | **n=256** | **0.06 ^a^** |
| Same day | 1007 (35.0%) | 908 (34.6%) | 99 (38.7%) |  |
| 1-7 days | 1091 (37.9%) | 1006 (38.3%) | 85 (33.2%) |  |
| 7-30 days | 531 (18.4%) | 490 (18.7%) | 41 (16.0%) |  |
| >30 days | 251 (8.7%) | 220 (8.4%) | 31 (12.1%) |  |
| **Median age in years (IQR)** | 36.4 (29.2, 44.7) | 36.3 (29.2, 44.7) | 38 (29.4, 45.4) | **0.19 ^b^** |
| **Age group (years)** | **n=2880** | **n=2624** | **n=256** | **0.56 ^a^** |
| 19-29 | 782 (27.2%) | 715 (27.2%) | 67 (26.2%) |  |
| 30-39 | 977 (33.9%) | 897 (34.2%) | 80 (31.3%) |  |
| 40-49 | 720 (25.0%) | 647 (24.7%) | 73 (28.5%) |  |
| 50+ | 401 (13.9%) | 365 (13.9%) | 36 (14.1%) |  |
| **Sex** | **n=2880** | **n=2624** | **n=256** | **0.46 ^a^** |
| Male | 1331 (46.2%) | 1207 (46.0%) | 124 (48.4%) |  |
| Female | 1549 (53.8%) | 1417 (54.0%) | 132 (51.6%) |  |
| **Marital Status** | **n=2868** | **n=2624** | **n=244** | **0.09 ^a^** |
| Single | 1341 (46.8%) | 1245 (47.4%) | 96 (39.3%) |  |
| Married or living with a partner | 1135 (39.6%) | 1026 (39.1%) | 109 (44.7%) |  |
| Separated/ Divorced | 149 (5.2%) | 132 (5.0%) | 17 (7.0%) |  |
| Widowed | 243 (8.5%) | 221 (8.4%) | 22 (9.0%) |  |
| **Education** | **n=2864** | **n=2624** | **n=240** | **0.52 ^a^** |
| Never went to school | 314 (11.0%) | 294 (11.2%) | 20 (8.3%) |  |
| Primary | 1176 (41.1%) | 1074 (40.9%) | 102 (42.5%) |  |
| Secondary or high school | 1087 (38.0%) | 991 (37.8%) | 96 (40.0%) |  |
| University | 287 (10.0%) | 265 (10.1%) | 22 (9.2%) |  |
| **Income** | **n=2786** | **n=2624** | **n=162** | **0.47 ^a^** |
| None | 991 (35.6%) | 934 (35.6%) | 57 (35.2%) |  |
| <50,000 CFA (<81.4 USD) | 1066 (38.3%) | 999 (38.1%) | 67 (41.4%) |  |
| 51-100,000 CFA (83.1–162.9 USD) | 423 (15.2%) | 397 (15.1%) | 26 (16.0%) |  |
| >100,000 CFA (>162.9 USD) | 306 (11.0%) | 294 (11.2%) | 12 (7.4%) |  |
| **HIV disease stage** | **n=2767** | **n=2624** | **n=143** | **0.90 ^a^** |
| Early (WHO stage 1 or 2) | 2019 (73.0%) | 1914 (72.9%) | 105 (73.4%) |  |
| Advanced (WHO stage 3 or 4) | 748 (27.0%) | 710 (27.1%) | 38 (26.6%) |  |
| Unknown/missing |  |  |  |  |
| **Depression** | **n=2873** | **n=2624** | **n=249** | **0.24 ^a^** |
| None (PHQ2 <3) | 2265 (78.8%) | 2076 (79.1%) | 189 (75.9%) |  |
| Depressive disorder (PHQ2 ≥3) | 608 (21.2%) | 548 (20.9%) | 60 (24.1%) |  |
| **Drinking** | **n=2866** | **n=2624** | **n=242** | **0.31^a^** |
| Never | 1091 (38.1%) | 1004 (38.3%) | 87 (36.0%) |  |
| Monthly | 1229 (42.9%) | 1129 (43.0%) | 100 (41.3%) |  |
| Weekly | 546 (19.1%) | 491 (18.7%) | 55 (22.7%) |  |
| **Smoking** | **n=2862** | **n=2624** | **n=238** | **0.44^a^** |
| Never | 2334 (81.6%) | 2145 (81.7%) | 189 (79.4%) |  |
| Former | 181 (6.3%) | 167 (6.4%) | 14 (5.9%) |  |
| Current | 347 (12.1%) | 312 (11.9%) | 35 (14.7%) |  |
| **Drug use** | **n=2857** | **n=2624** | **n=233** | **1.00^a^** |
| Never | 2847 (99.6%) | 2614 (99.6%) | 233 (100%) |  |
| Ever | 10 (0.4%) | 10 (0.4%) | 0 (0%) |  |

**^a^ Chi squared test; ^b^ Kruskal-Wallis Test**
